# Prevalence of HAV Ab, HEV (IgG), HSV2 IgG, and Syphilis Among Sheltered Homeless Adults in Tehran, 2012

**DOI:** 10.15171/ijhpm.2017.74

**Published:** 2017-07-08

**Authors:** Fatemeh Jahanbakhsh, Fahimeh Bagheri Amiri, Abbas Sedaghat, Noushin Fahimfar, Ehsan Mostafavi

**Affiliations:** ^1^Virology Research Group, Pasteur institute of Iran, Tehran, Iran.; ^2^Urology and Nephrology Research Center, Shahid Beheshti University of Medical Sciences, Tehran, Iran.; ^3^Blood Transfusion Research Center, High Institute for Research and Education in Transfusion Medicine, Tehran, Iran.; ^4^Center for Disease Control, Ministry of Health and Medical Education, Tehran, Iran.; ^5^Department of Epidemiology and Biostatistics, School of Public Health, Tehran University of Medical Sciences, Tehran, Iran.; ^6^HIV/STI Surveillance Research Center, and WHO Collaborating Center for HIV Surveillance Institute for Futures Studies in Health, Kerman University of Medical Sciences, Kerman, Iran.; ^7^Department of Epidemiology and Biostatistics, Research Centre for Emerging and Reemerging Infectious Diseases, Pasteur Institute of Iran, Tehran, Iran.

**Keywords:** Hepatitis A Virus (HAV), Hepatitis E Virus (HEV), Herpes Simplex Virus Type 2 (HSV2), Syphilis, Homeless, Iran

## Abstract

** Background:** This study investigated the prevalence for hepatitis A virus (HAV), hepatitis E virus (HEV), herpes simplex virus type 2 (HSV2) and syphilis among homeless in the city of Tehran.

**Methods:** In this cross-sectional study, 596 homeless were recruited in Tehran. A researcher-designed questionnaire was used to study demographic data. Using enzyme-linked immunoassay, and rapid plasma reagin (RPR) test, we evaluated the seroprevalence of HAV anti-body, HEV IgG, herpes, HSV2 IgG, and syphilis among sheltered homeless in Tehran. The associations between the participant’s characteristics and infections were evaluated using logistic regression and chi-square.

**Results:** A total of 569 homeless, 78 women (13.7%) and 491 men (86.3%) were enrolled into the study from June to August 2012. Their age mean was 42 years and meantime of being homeless was 24 months. Seroprevalence of syphilis, HEV IgG, HSV2 IgG and HAV Ab was 0.55%, 24.37%, 16.48%, and 94.34%, respectively. History of drug abuse was reported in 77.70%; 46.01% of them were using a drug during the study and 26.87% of them had history of intravenous drug abuse. Among people who had intravenous drug abuse, 48.25% had history of syringe sharing.

**Conclusion:** The prevalence of HAV, HEV and HSV2 were higher than the general population while low prevalence of syphilis was seen among homeless peoples who are at high risk of sexually transmitted infection (STD). Our findings highlighted that significant healthcare needs of sheltered homeless people in Tehran are unmet and much more attention needs to be paid for the health of homeless people.

## Introduction

Homeless includes people who have no place to sleep and sleep in public or private shelters. They are including mental health disorders, alcoholics^1^ and in Iran, mostly injecting drug users, and immigrants.^2^ The life expectancy of homeless people is much shorter than the general population, and rates of infectious diseases are higher among them.^3^ High risks of infectious diseases among homeless people are related to their living condition, poor sanitation and living within the group that makes them susceptible to many communicable diseases, outbreaks of HIV, tuberculosis, and viral hepatitis have been reported among them in many countries.^1,3,4^ Homeless people are also at higher risk than the general population for viral hepatitis and syphilis due to social and behavioral factors that influence the occurrence of these diseases.^5-7^

Homeless people are at high risk for viral hepatitis (A, B, and C) because their lifestyles might include injection drug use (IDU) and poor hygiene, but data on hepatitis E virus (HEV) and hepatitis A virus (HAV) prevalence among them are limited.^7-9^

Syphilis and herpes simplex virus type 2 (HSV2) are common sexually transmitted infections (STDs).^10,11^ *Treponema pallidum* infects at least 12 million persons annually. HSV2 seropositive persons have a lifelong risk of infecting their sexual partners.^12^ Genital ulcer disease due to both syphilis and HSV2 is associated with an increased risk of obtaining HIV.^11^ Findings of a previous study among homeless people of this study showed a high prevalence of HIV among them.^7^

There is no precise estimate on the number of homeless people and the rate of their risky behaviors in Iran. Due to a lack of updated information on infectious diseases, and the absence of a study on the situation of infectious diseases among homeless people in Iran, the aim of this study was to investigate the prevalence of HAV, HEV, HSV2 and syphilis among sheltered adult homeless people in Tehran and to evaluate the high-risk behaviors associated with these infections among them.

## Methods

This study was conducted in Tehran, the capital of Iran, from June to August 2012.

Participants were recruited from five centers working under the authority of the municipality. Homeless people were eligible to participate in the study if they were 18–60 years old and had been continuously or discontinuously homeless (during the month prior to the study period) for at least 10 days.^13^

A homeless person was defined as someone who had no home or shelter to reside in, and instead resided on the corners of streets, in parks, or in public places, if there was no designated residence provided by governmental or non-governmental organizations.

In this study, verbal informed consent was provided. This form included descriptions of voluntary participation and the incentives for participating in the study. A researcher-made questionnaire was used to assess the behaviors.

Blood samples were tested to detect HAV antibody (Ab), hepatitis E IgG (HEV IgG) and HSV2 IgG, using commercially available ELISA kits (Dia.Pro Diagnostic BioProbes srl, Italy) and rapid plasma reagin (RPR) test for syphilis.

Data analysis was carried out by SPSS (version 16, SPSS Inc., Chicago, IL, USA) software package. Associations between participants’ characteristics and the infections were evaluated using logistic regression and chi-square test. *P* values less than 0.05 were considered statistically significant.

## Results

A total of 569 homeless, 78 women (13.08%) and 491 men (82.38%) were enrolled into the study from five centers in Tehran during June to August 2012. Their age mean was 42 years and meantime of being homeless was 24 months. History of drug abuse among homeless people was 77.70%, 46.01% of them were using a drug during study and 26.87% of them had history of intravenous drug abuse. Among people who had intravenous drug abuse, 48.25% had history of syringe sharing.

Seroprevalence of syphilis (among 542 tested homeless) was 0.55%, HEV IgG (among 562 tested homeless) was 24.37%, HSV2 IgG (among 546 tested homeless) was 16.48%. History of sexual contact (legal or illegal) was seen among 80.00% of‏ the participants; of these, only 33.86% used condom in last sexual contact. History of either selling sex or having sex with other men was seen among 14 women (21.87%) and 36 men (9.30%), respectively.

Univariate analysis showed no significant correlation between seroprevalence of syphilis and HSV-2. There was a significant positive association between HAV, HEV seroprevalence and age. Prevalence of HAV significantly was higher in men. Education level had significant relation with HEV; HEV prevalence among people with higher level of education was lower than those who have low education (Table).

**Table:** Univariate Analysis of Factors Associated With the Prevalence of HAV, HEV, HSV2 and Syphilis on Homeless People in Tehran, 2012

**Variables**	**Categories**	**RPR**	**OR (95% CI)**	**HAV**	**OR (95% CI)**	**HEV**	**OR (95% CI)**	**HSV2**	**OR (95% CI)**
Age*	More than 42 year	261 (1.15)	0.61 (0.02-8.07)	258 (98.06)	5.44 (5.05-14.39)	265 (35.47)	3.27 (2.16-4.96)	260 (16.54)	1.04 (0.65-1.64)
	Less than 42 years	269 (0.00)		268 (90.30)		285 (14.39)		274 (16.06)	
Gender	Female	78 (0.00)	0.61 (0.02-8.07)	78 (84.62)	4.22 (1.96-9.09)	484 (25.62)	1.72 (0.92-3.23)	468 (15.60)	0.66 (0.37-1.20)
	Male	464 (0.64)		460 (95.87)		78 (16.17)		78 (21.79)	
Educational level	Illiterate	65 (0.00)	-	66 (100.00)	-	67 (40.30)	Reference	66 (16.67)	Reference
	Literate	28 (0.00)	-	28 (100.00)	-	28 (46.43)	1.28 (0.53-3.12)	28 (21.43)	1.36 (0.45-4.14)
	Primary school	105 (1.90)	-	103 (95.15)	-	110 (30.91)	0.66 (0.35-1.25)	105 (19.05)	1.18 (0.52-2.65)
	Secondary school	142 (0.00)	-	143 (95.10)	-	149 (18.12)	0.33 (0.17-0.62)	146 (14.38)	0.84 (0.38-1.86)
	High school	143 (0.70)	-	139 (90.45)	-	143 (16.78)	0.30 (0.16-0.58)	139 (15.11)	0.89 (0.40-1.97)
	Academic level	44 (0.00)	-	43 (86.05)	-	48 (16.67)	0.30 (0.12-0.73)	46 (15.22)	0.90 (0.32-2.52)
Duration of being Homeless^a^	More than 24 months	233 (0.43)	0.61 (0.02-8.07)	232 (93.66)	0.99 (0.48-2.07)	240 (25.83)	1.16 (0.78-1.72)	232 (15.94)	0.91 (0.57-1.45)
	Less than 24 months	285 (0.70)		282 (93.97)		298 (23.15)		290 (17.24)	
History of Drug use	Yes	412 (0.73)	4.88 (0.42-163.20)	409 (94.13)	1.05 (0.44-2.50)	433 (23.56)	1.26 (0.81-1.98)	417 (15.35)	1.38 (0.83-2.30)
	No	126 (0.00)		125 (94.40)		125 (28.00)		125 (20.00)	
Drug use (currently)	Yes	180 (0.56)	0.64 (0.02-8.51)	175 (92.00)	1.95 (0.84-4.50)	200 (24.00)	0.96 (0.61-1.49)	185 (18.92)	0.61 (0.36-1.05)
	No	232 (0.86)		234 (95.73)		233 (23.18)		232 (12.50)	
**Kind of Drug Use**									
Hashish	Yes	11 (0.00)	25.11 (0.51-1274)	10 (100.00)	1.43 (0.17-42.76)	11 (45.45)	2.83 (0.82-9.73)	10 (10.00)	0.46 (0.06-3.76)
	No	169 (0.59)		165 (91.51)		189 (24.34)		175 (19.42)	
Kerack^b^	Yes	41 (0.00)	5.54 (0.11-266.30)	40 (90.00)	0.72 (0.21-2.43)	53 (22.64)	0.90 (0.43-1.90)	43 (16.28)	0.79 (0.32-1.97)
	No	139 (0.72)		135 (92.59)		147 (24.29)		142 (19.72)	
Heroin	Yes	60 (0.00)	3.27 (0.07-156.60)	57 (92.98)	1.23 (0.37-4.10)	67 (28.36)	1.42 (0.73-2.78)	60 (18.33)	0.95 (0.43-2.08)
	No	120 (0.83)		118 (93.22)		133 (21.80)		125 (19.20)	
Methamphetamine	Yes	80 (1.25)	2.04 (0.04-97.87)	75 (92.00)	1.01 (0.34-3.05)	90 (20.00)	0.66 (0.34-1.28)	80 (22.50)	1.60 (0.76-3.37)
	No	99 (0.00)		99 (91.92)		109 (27.52)		104 (15.38)	
Opium	Yes	60 (0.00)	0.83 (0.02-36.63)	57 (94.74)	1.85 (0.50-6.91)	70 (22.86)	0.91 (0.46-1.80)	60 (13.33)	0.56 (0.24-1.32)
	No	120 (0.83)		118 (90.68)		130 (24.42)		125 (21.60)	
Others	Yes	11 (0.00)	0.11 (0.01-5.48)	10 (90.00)	0.77 (0.09-6.56)	12 (41.67)	2.41 (0.73-7.97)	11 (90.90)	0.41 (0.05-3.33)
	No	169 (0.59)		165 (92.12)		188 (22.87)		174 (19.54)	
Injecting drugs	Yes	303 (0.33)	0.54 (0.01-25.60)	101 (93.07)	0.87 (0.35-2.14)	113 (25.66)	1.10 (0.67-1.81)	103 (15.53)	0.89 (0.48-1.64)
	No	99 (0.00)		297 (93.94)		310 (23.87)		304 (17.11)	
Sharing needle	Yes	49 (0.00)	-	51 (92.16)	0.76 (0.14-3.91)	58 (29.31)	1.45 (0.62-3.41)	52 (13.46)	0.71 (0.24-2.08)
	No	49 (0.00)		49 (93.88)		54 (22.22)		50 (18.00)	
Age at the start of injecting drug		43 (0.00)	-	44 (95.45)	1.79 (0.31-10.26)	49 (26.53)	1.04 (0.44-2.46)	44 (15.91)	1.06 (0.35-3.21)
		50 (0.00)		51 (92.16)		58 (25.86)		53 (15.09)	
History of Incarceration	Never	273 (1.09)	-	271 (93.36)	Reference	281 (25.53)	Reference	273 (17.94)	Reference
	More than 10 years ago	64 (0.00)	-	62 (96.77)	2.13 (0.48-9.45)	65 (24.61)	0.97 (0.52-1.81)	62 (17.74)	0.99 (0.48-2.03)
	Recent 10 years	194 (0.00)	-	194 (94.33)	1.18 (0.55-2.57)	205 (23.41)	0.90 (0.59-1.38)	200 (14.00)	0.74 (0.45-1.23)
Duration of Incarceration (in last 10 years)^a^	Upper than 11 months	95 (0.00)	-	95 (93.68)	0.79 (0.24-2.71)	101 (28.71)	1.88 (0.97-3.66)	98 (13.27)	0.88 (0.39-1.95)
	Lower than 11 months	98 (0.00)		98 (94.90)		102 (17.65)		101 (14.85)	
History of having sex	Yes	429 (0.47)	0.48 (0.04-5.37)	424 (93.63)	0.58 (0.20-1.70)	441 (24.72)	1.09 (0.67-1.77)	429 (16.62)	0.77 (0.45-1.32)
	No	104 (0.96)		105 (96.19)		112 (23.21)		108 (19.44)	
Condom use at last sexual encounter	Yes	145 (0.00)	1.28 (0.04-19.87)	141 (90.07)	0.44 (0.20-0.96)	147 (22.45)	0.82 (0.52-1.31)	144 (12.50)	0.68 (0.38-1.22)
	No	282 (0.71)		281 (95.37)		292 (26.03)		283 (17.31)	
Sex work (female)	Yes	14 (0.00)	-	14 (78.57)	2.14 (0.46-9.90)	14 (7.14)	3.90 (0.46-32.94)	14 (14.28)	1.26 (0.24-6.61)
	No	52 (0.00)		53 (88.68)		52 (23.08)		52 (17.31)	
Men who have sex with men	Yes	32 (0.00)	6.64 (0.20-104.60)	32 (87.50)	3.34 (1.02-10.93)	33 (24.24)	1.06 (0.46-2.44)	32 (18.75)	0.72 (0.28-1.85)
	No	321 (0.62)		317 (95.90)		332 (25.30)		322 (14.29)	

Abbreviations: HAV, hepatitis A virus; HEV, hepatitis E virus; HSV2, herpes simplex virus type 2. ^a^ Median of the variable is used for analysis; ^b^ A purified and potent form of heroin (not to be mistaken with crack cocaine).

## Discussion

Finding of this study showed moderately high seroprevalence of HEV (24.37%) and high seroprevalence of HAV amongst homeless people of Tehran, with almost near to all of participants (94.34%) having HAV IgG. Although syphilis seroprevalence in this study was not high (0.55%.), nevertheless, the high prevalence of IDUs (20.73%), and needle sharing (10.72%) between Tehran homeless people can serve as a warning for future blood borne epidemics among homeless people who are also intravenous drug users.

Sexually transmitted infections (STIs) that cause open sores on the genitals, like herpes and syphilis can increase HIV transmission.^14^ HSV-2 increases susceptibility to HIV infection through physical disruption of the epithelial surface by HSV-2, and recruitment and persistence of inflammatory cells in the genital tract during HSV-2 reactivation at mucosal surfaces.^14^ Genital herpes, caused by the HSV-2, is one of the most common sexually transmitted infections worldwide. Prevalence of HSV-2 IgG in northern Iran was reported 3.5% among general population, and significant correlation was seen between age, marital status, job, symptoms, and history of disease and HSV-2 seroprevalence.^15^ Surprisingly, in contrast with Noell et al study,^8^ there was no correlation between inconsistent condom use and HSV2 seroprevalence in this study. The prevalence of HSV2 among homeless people in the present study is higher than its prevalence in the general population, even higher than the prevalence reported by other studies.^9,10^ This can be attributed to the poor sexual health of the targeted homeless in compare with the general population which is line with higher prevalence of HIV prevalence among them.^7^

The overall prevalence of syphilis is relatively low in Iran. The reported prevalence in the most studies was limited to 0.2% to 0.6%.^16^ The prevalence of syphilis in blood donors in Tehran province between 2005 to 2011 was reported 10.5 per 100 000 donation.^17^ The findings of studies among adolescence and street children in Tehran,^18-20^ are compatible with the findings of the present study, three individuals out of 542 sera had reactive RPR test and seroprevalence of syphilis was 0.55%. The prevalence of syphilis among the other high-risk group like female sex-worker in Kerman, south-east Iran and Shiraz was 7.2% and 0.278% respectively.^21,22^ The high prevalence of HIV,^7^ HSV2 and low prevalence of syphilis in the present study and overall in Iran indicates that syphilis infection is not prevalent in Iran, even among high-risk population. Prevalence of syphilis in Brazil was reported 7.0% among homeless.^23^ No significant association was found between high-risk sexual behavior and syphilis. Sheltered homeless people in Tehran are living in a situation relatively similar to prisons. Overcrowded sheltered, low personal and environment hygiene and exposed to high-risk behaviors, all together make them vulnerable to infectious disease, particularly sexually transmitted disease. Although syphilis seems not to be significant infectious diseases among homeless in Tehran but due to high rate of sexual and drug-related behaviors, they may be at a higher risk in the future. Even though the prevalence of syphilis is low among the studied people, the surveillance of this disease should be continued throughout the country.

Among the study population 94.34% were found HAV seropositive. HAV is an endemic disease in Iran and is highly prevalent across the country. Its seroprevalence in the general population of Tehran^24^ and Fars^25^ was reported 90% and 88.2%, respectively. Similar to this study, HAV seroprevalence was higher among men and older ages. Other anti-HAV seroprevalence studies among homeless people conducted in Australia, Canada and San Francisco in the United States found similarly high anti-HAV prevalence of 48%, 53% and 2%, respectively. Like the present study, these studies found older age to be a major predictor of anti-HAV.

This work showed that the seroprevalence of HEV was moderately high (24.37%) in homeless people. Similar to the studies previously have done in Iranian general population on blood donors, the prevalence of HEV IgG in homeless persons was lower among younger people with higher level of education^24,26^ and no differences in gender. To the best of our knowledge, seroprevalence of HEV in Iranian homeless has never been reported up to now. Based on this study, the HEV seroprevalence in Iranian homeless was much higher than what have seen in Los Angeles (13.6)^27^ and Marseille in France (11.6%).^7^ HEV seroprevalence in general population of Tehran (9.3%)^24^ and Isfahan (3.8%)^26^ was much lower than this study. The prevalence of HAV and HEV in this study is higher than the prevalence observed in the general population of the country. This finding suggests that homelessness increases the risk of being HAV and HEV positive.

Several limitations in this study must be noted. First, all data are from self-reports. Second, because of lifestyles that are extremely transient and sometimes cryptic, it is impossible to obtain a truly representative sample of “homeless” and generalizations to all homeless populations are not possible. However, the relatively large size of the sample gives some confidence that these data are applicable to the more visible part of the homeless population in Tehran. Third, in this study RPR test was used to screen sera for Syphilis. RPR test is a non treponemal antibody test, although these screening tests are non-specific, it has traditionally been used for initial syphilis screening due to their relative low-cost and ease of performance. The other restriction is lack of enough samples for all tests in some cases. Since this study was conducted after our main study, to check the vulnerability of homeless people to HIV, Tuberculosis and viral hepatitis, some of the blood samples were not adequate for present study.^13^ Actually present study was a secondary data analysis; therefore, questioner and sampling have not designed for these disease and tests. Accordingly, some questions and high-risk behavior which could be helpful in the final analysis have not been asked.

Another limitation of the study was that pathogen infection was determined based on seropositivity to IgG antibodies. IgG antibodies reflect prior infection, but are not sensitive indicators of current infection. For instance, it is likely the high sero-prevalence of HAV reflect exposure to this virus in early childhood (as it is common in most low and middle-income settings), rather than current infection, and as such not a risk for potential onward transmission. A similar thing may be said with regards to syphilis, as low titers of RPR may be remained for long periods (or be prone to false positive results). Unfortunately, our data are unable to address this issue. Notably, IgG antibodies were used to define infection in most studies among homeless people.^4,6,7,13,19,23^

In conclusion, overcrowded sheltered, poor living conditions and limited access to healthcare systems, exposes homeless persons to communicable infections, which may spread among them and lead serious public health concerns. The risks of infectious disease outbreaks in homeless populations are significantly higher than those in the general population. These increased risks are a public health challenge for the population as a whole. Implementation of specific strategies to reduce these risks is crucial. According to the result of this study, the necessity of implementing preventive programs is recommended. Educational programs, counseling and testing of transmission of infection diseases and sexually transmitted diseases should be included in the centers provided for homeless people.

## Acknowledgments

The research team is thankful of research committee of the Pasteur Institute of Iran, Tehran, Iran which approved this project (No. 12286) and the Centre for Communicable Disease Control in the Ministry of Health and Medical Education (MoHME), Tehran, Iran, which supported this project.

## Ethical issues

The research protocol was reviewed by the Ethical Committee of Pasteur Institute of Iran, Tehran, Iran and was exempt from full review.

## Competing interests

Authors declare that they have no competing interests.

## Authors’ contributions

FJ, FBA, and EM obtained the data, performed analyses, and wrote results. NF, EM, and AS took a lead in review of literature on emergency preparedness and EM performed literature review for health informatics. All five authors contributed to discussion and conclusions.

## Authors’ affiliations

^1^Virology Research Group, Pasteur institute of Iran, Tehran, Iran. ^2^Urology and Nephrology Research Center, Shahid Beheshti University of Medical Sciences, Tehran, Iran. ^3^Blood Transfusion Research Center, High Institute for Research and Education in Transfusion Medicine, Tehran, Iran. ^4^Center for Disease Control, Ministry of Health and Medical Education, Tehran, Iran. ^5^Department of Epidemiology and Biostatistics, School of Public Health, Tehran University of Medical Sciences, Tehran, Iran. ^6^HIV/STI Surveillance Research Center, and WHO Collaborating Center for HIV Surveillance Institute for Futures Studies in Health, Kerman University of Medical Sciences, Kerman, Iran. ^7^Department of Epidemiology and Biostatistics, Research Centre for Emerging and Reemerging Infectious Diseases, Pasteur Institute of Iran, Tehran, Iran.

## Key messages

**Implications for policy makers**

- Homeless people are those who have no place to sleep and sleep in public or private shelters. They are including mental health disorders, alcoholics and mostly injecting drug users, and immigrants.
- Homeless people are also at higher risk than the general population for viral hepatitis and syphilis due to social and behavioral factors that influence the occurrence of these diseases.
- Overcrowded shelters, poor living conditions and limited access to healthcare systems, expose homeless persons to communicable infections, which may spread among them leading serious public health concerns.

**Implications for public**

Our findings highlighted that significant healthcare needs of sheltered homeless people in Tehran are unmet and much more attention needs to be paid for the health of homeless people. The risks of infectious disease outbreaks in homeless populations are significantly higher than those in the general population. These increased risks are a public health challenge for the population as a whole. Implementation of specific strategies to reduce these risks is crucial.
